# Commentary: Health and safety practices and policies concerning human exposure to RF/microwave radiation

**DOI:** 10.3389/fpubh.2026.1752150

**Published:** 2026-02-19

**Authors:** William H. Bailey, Kenneth R. Foster, Chung-Kwang Chou

**Affiliations:** 1 Health Sciences, Exponent, Bowie, MD, United States; 2 Department of Bioengineering, University of Pennsylvania, Philadelphia, PA, United States; 3 C-K Chou Consulting, Dublin, CA, United States

**Keywords:** exposure limits, ICNIRP, IEEE, microwave, radio frequency, WHO-EMF systematic reviews

## Introduction

Lin (1) comments about “health and safety practices” concerning human exposure to radio frequency (RF) energy and two major international exposure limits [IEEE C95.1-2019 (2) and ICNIRP 2020 (3)]. Lin's commentary is misleading and inaccurate on important topics. First, it misrepresents the IEEE C95.1-2019 (2) and ICNIRP (2020) limits (3). Second, it fails to distinguish between biological effects and health hazards, with several examples incorrectly presented. Third, his critique of World Health Organization (WHO) sponsored systematic reviews (SRs) relies on selected criticisms without evaluating their objectivity and substance. Consequently, his commentary lacks an adequate basis for its conclusions.

## Discussion

1. Referring to Table 1 (1), Lin states “*the exposure limits allow tissue temperature rises in the human head, limbs, and torso to as high as* 5 °C.” This statement is incorrect because 5 °C is the threshold for adverse effects on the body surface for 6 −300 GHz local exposures; the exposure limits will not allow a temperature increase to 5 °C, although Lin explained there are safety or reduction factors of 2 and 10 near the end of the paragraph, and called them marginal. ICNIRP has limits of 2 °C for head and torso tissues and 5 °C for limbs for local exposures below 6 GHz; above 6 GHz, the absorbed power density limits in columns 8, 10 are for all body surface tissues, not separated as Local head-torso and Local limbs as in Lin's Table 1. The averaging area for 30–300 GHz is 1 cm^2^ and not 2 cm^2^. The exposure limits in W/m^2^ are stated correctly in the text but the units are listed incorrectly as W/cm^2^ in Lin's Table 1, which is 10,000 higher. Lin's discussion of Dagro et al. (4) also has errors on the power density units. The units cited by Lin are off by a factor of 1 million.
2. Lin criticizes that there is no scientific rationale for IEEE to adopt a local SAR limit of 2 W/kg averaged over a 10-g mass, other than harmonization with the ICNIRP. The IEEE Standard C95.1-2005 and 2019 (2) both provide this rationale in an extensive discussion [see B.4.1.2.2. and B.7.5 in the 2019 revision (2)]. Harmonization of limits and frequency ranges was an important part of the process as long as justified based on scientific reasoning.
3. Lin argues that the microwave auditory effect is adverse but occurs at exposure levels allowed by guidelines. The IEEE C95.1-2019 standard (2) and ICNIRP (2020) guidelines (3) explicitly state that the limits are designed to protect against adverse health effects, identified as “established” adverse health effects (IEEE) (2) and “substantiated” adverse health effects” (ICNIRP, 2020) (3) of exposures to electric fields, magnetic fields, and electromagnetic fields. They were not set on the basis of speculative hazards or biological data that are unrelated to health. Both standards have evaluated and explicitly excluded this effect. The IEEE C95.1-2019 standard (2) states “*The phenomenon of RF hearing in humans is a well-established biological effect with no known adverse health consequence. The RF-induced sounds are similar to other common sounds*.” ICNIRP 2020 also does not include limits for auditory effects for similar reasons (3).
4. Lin alleges “[*t]he applicability of the limits for safe long-term exposure to low-level RF radiation is questionable*” and “*are flawed and are not applicable to long-term, low-levels exposures*.” While that is his opinion and shared by others, his cited support consists of one review “(18)” and 7 opinion pieces “(17–22, 33)”. IEEE C95.1-2019 in Section 1.3.1 rebuts these opinions:

The literature review also evaluated the possibility of adverse health effects associated with chronic low-level exposure. For exposures to electric, magnetic, and electromagnetic fields at frequencies between 0 Hz and 300 GHz, the following two conclusions were reached:

a) The weight-of-evidence provides no credible indication of adverse effects caused by chronic exposures below levels specified in this standard.

b) No biophysical mechanisms have been scientifically validated that would link chronic exposures below levels specified in this standard to adverse health effects (2).

ICNIRP has similarly addressed this issue in its guideline (3) and ICNIRP 2020 Q&A (https://www.icnirp.org/en/rf-faq/index.html). More systematic and in depth research is needed to address the range of published opinions, including assessments of risk of bias which are notably lacking in the sources that Lin relies on for his conclusions.
5. Lin criticizes IEEE and ICNIRP exposure limits “*as grounded on a strong RF heating conviction—only RF heating effects revealed by measurable tissue temperature rises.”* Neither IEEE and ICNIRP nor health agencies have identified “substantiated adverse health effects” with thresholds below thermally-based limits after evaluating exposure across the entire range of RF levels in the literature. This is far different from asserting that the standards considered “only RF heating effects” exist. For example, ICNIRP 2020 Q&A states:

*ICNIRP considers all potential adverse health effects, and sets restrictions to ensure that none occur, regardless of the mechanism of interaction between exposure and the body. The lowest exposure levels that can cause adverse health effects are due to thermal mechanisms, and so restrictions have been set based on the thermal effects, as these will protect against any other effects that could occur at higher exposure levels*.

ICNIRP (5) recently reviewed the literature further and identified data gaps in the literature but did not identify reliable evidence for effects of RF exposure that would presently warrant a change in its guidelines. The IEEE International Committee on Electromagnetic Safety website cites reviews by expert groups and health agencies around the world in the last 15 years; none reported consistent, confirmed health effects at levels below IEEE and ICNIRP exposure limits (https://sagroups.ieee.org/ices/expert-reviews/).
6. Lin criticizes some of the 12 recent systematic reviews (SR) of various RF/health issues supported by the World Health Organization (6) that “*appear to be biased with strong conviction of nothing but heat to worry about with RF microwave radiation*” (1). This misrepresents actual conclusions of the reviews; they are more detailed and cannot be summarized as stated. Rather than dismissing non-thermal effects reviewed, conclusions focused on the poor overall quality of the studies reviewed, which prevented drawing conclusions with high confidence. Several SRs provided detailed recommendations for improved follow-up research to address these weaknesses. Evaluation of this complex scientific literature includes consideration of risk of bias, quality of study design, possible study-selection bias, and certainty of evidence. These complex issues cannot be adequately addressed in commentaries by Lin or us, and await more comprehensive multidisciplinary assessments of SRs, all commentaries, and more publications in planned reports by scientific and health agencies including the WHO Task Group in the upcoming EHC Monograph of RF fields, and by the International Agency for Research on Cancer (IARC). Lin cites Mevissen et al. (7) for reporting a high certainty of evidence for links between RF fields and two cancers. A letter from Karipidis et al. (8) and a response by Mevissen et al. (9) appeared after Lin's commentary and the preparation of this present commentary.
